# Affect and Cognition in Managerial Decision Making: A Systematic Literature Review of Neuroscience Evidence

**DOI:** 10.3389/fpsyg.2022.762993

**Published:** 2022-03-09

**Authors:** Matteo Cristofaro, Pier Luigi Giardino, Andrea P. Malizia, Antonio Mastrogiorgio

**Affiliations:** ^1^Department of Management and Law, University of Rome ‘Tor Vergata’, Rome, Italy; ^2^Doctoral School of Social Sciences, University of Trento, Trento, Italy; ^3^Molecular Mind Laboratory (MoMiLab), IMT School for Advanced Studies Lucca, Lucca, Italy; ^4^Laboratory for the Analysis of CompleX Economic Systems (AXES), IMT School for Advanced Studies Lucca, Lucca, Italy

**Keywords:** affect, cognition, decision making, neuroscience, Systematic Literature Review (SLR), behavioral strategy

## Abstract

How do affect and cognition interact in managerial decision making? Over the last decades, scholars have investigated how managers make decisions. However, what remains largely unknown is the interplay of affective states and cognition during the decision-making process. We offer a systematization of the contributions produced on the role of affect and cognition in managerial decision making by considering the recent cross-fertilization of management studies with the neuroscience domain. We implement a Systematic Literature Review of 23 selected contributions dealing with the role of affect and cognition in managerial decisions that adopted neuroscience techniques/points of view. Collected papers have been analyzed by considering the so-called reflexive (X-) and reflective (C-) systems in social cognitive neuroscience and the type of decisions investigated in the literature. Results obtained help to support an emerging “unified” mind processing theory for which the two systems of our mind are not in conflict and for which affective states have a driving role toward cognition. A research agenda for future studies is provided to scholars who are interested in advancing the investigation of affect and cognition in managerial decision making, also through neuroscience techniques – with the consideration that these works should be at the service of the behavioral strategy field.

## Introduction

Since Simon’s (1947) *administrative man* – featured by bounded rationality – and the conceptualization of a firm’s performance as the result of the decision makers’ collective choice (Cyert and March, 1963) – a series of studies in management studies advanced the debate of how organizational actors (individually or collectively) make decisions (Kahneman, 2011; Lovallo and Sibony, 2018; Cristofaro, 2021a). Over the years, managerial decision making – concerning the decisional activities made at the low-, middle-, and top-management levels (Koontz et al., 1980) – attracted the interest of scholars in various areas, mainly due to its cross-disciplinary nature (Cristofaro, 2017; Adinolfi, 2021). A great advancement has been made by the *Behavioral Decision Theory* (BDT), that originated in the ‘60s for the study of the *real* behavior of people when making decisions (Edwards, 1961), and then increasingly adopted/developed in managerial decision-making research stimulating reflections on bounded rationality (e.g., Edwards, 1961; March, 1978; Kahneman, 2011; Powell et al., 2011; Sibony et al., 2017; Abatecola et al., 2021; Cristofaro and Giannetti, 2021).

From the progress made, the role of affective states has continuously and increasingly gained momentum within managerial decision-making research (e.g., Cristofaro, 2017, 2019, 2021a,b). This happened mainly because affective states are considered the first biological reaction to stimuli in a decisional environment, condensing all other irrational impulses (Weick, 1979). Emotional response, however, may not only directly influence the initiation and/or the output of a decision path, but they can also influence the content and depth of thought within decision-making processes at the individual and collective levels (e.g., Damasio, 1994; Lerner et al., 2013; Cristofaro, 2019).

However, as reported by the Call for Papers on the research topic “Affect and Cognition in Upper Echelons’ Strategic Decision Making: Empirical and Theoretical Studies for Advancing Corporate Governance” to which this article contributes, what remains largely unknown in managerial decision-making research is “the interplay of affective states and cognition; considered by some scholars to be two parallel competitive systems of the human mind”. From that, we want to answer the following research question: *how do affect and cognition interact in managerial decision making?* Concerning the definition of affect, we can recall the contribution brought by Forgas (1995), who asserted that this word depicts a broad array of various affective states (the main term we will use hereafter), among which the preeminent ones are “moods” and “emotions”. Specifically, moods are related to low intensive and lasting affective circumstances (e.g., feeling down) that cannot be identified as a reaction to a precedent situation, while emotions, on the contrary, group all those affective reactions into a specific event. With regard to the definition of cognition, Neisser (1967) has defined it as the mental procedure through which inputs, such as information, are transformed, reduced, elaborated, and gathered, and then put into practice when needed.

To answer this study’s research question in a solid and “new” way, we offer a first systematization of contributions on managerial decision making that implement neuroscience techniques/points of view; thus allowing this review to robustly inform the affect-cognition debate. In this regard, a series of position papers/reviews/commentaries have been published about the impact of neuroscience approach and techniques in management studies (e.g., Becker et al., 2011; Powell, 2011; Ashkanasy et al., 2014; Ward et al., 2015; Jack et al., 2019; Massaro et al., 2020; Cucino et al., 2021), but only a few looked at the decision-making processes of managers within organizations. The sole contribution that tried to look at the influence of neuroscience studies in managerial decision making was Butler et al. (2016); however, for their results categorized under the “organizational behavior cluster” heading, some of the 12 contributions in that sample did not deal with managerial decision-making processes (e.g., Peterson et al., 2008), as well as it was not focused on shedding light on the affect-cognitive debate.

The collected 23 papers have been thematically analyzed by considering the type of managerial decisions, and under the reflexive (X-) and reflective (C-) systems of our brain, which seem to be devoted differently in describing consciousness, awareness, and mental processes (Lieberman et al., 2002; Lieberman, 2007; Lieberman-Aiden et al., 2009). From the analysis, affect and cognition work in a meaningful interplay that directs decision-making processes of managers, with affective states having an initial (but not exclusively) driving role. This result shed light on the possibility of a “unified” mind processing theory (Sadler-Smith, 2016; Cristofaro, 2020a; Cristofaro and Giannetti, 2021) for which the two Systems of our mind – System 1, devoted to operating mental processes that are fast and automatic, and System 2, devoted to operating mental processes that are consciously monitored (Kahneman, 2003) – are not in conflict, but they operate dialectically. These are the main theoretical implications of this paper. Yet, the main implications for research coming from this paper are related to the need for: *(i)* continuing the deconstruction of the distinction between irrational/automatic vs. rational/deliberate; *(ii)* articulating the ecological dimension of decision-making; and *(iii)* trying distinguishing the brain default activity by the task-evoked one, occurring while performing decision tasks.

## Theoretical Background

### Affect and Cognition in Decision Making

Since the birth of the bounded rationality concept by Simon (1947), scholars have been increasingly involved in identifying how management decisions are made. This endeavor has found fertile ground in *Behavioral Decision Theory* (BDT).

*Behavioral Decision Theory* was born to understand *real* human behavior in decision making by studying, for example, models for static risky decision making, utility function, subjective probability, variance preferences, and personality variables, mainly through experimentations and computational models (Edwards, 1961). In particular, BDT tries to explain why decision makers go beyond normative assumptions, such as violating expected utility axioms (Einhorn and Hogarth, 1981). In this regard, initial progress of BDT studies (e.g., Good, 1962) lead to depict rationality being shaped by unconscious psychological events and external forces that determine human decisions and their consistency. This updated conceptualization of rationality in organizations, rooted in Simon (1947), stimulated other new theories within BDT. The main contribution in this direction was the *Behavioral Theory of the Firm* by Cyert and March (1963), which stated that decisions in organizations are always made in the presence of scarce information and negotiated within coalitions composed of managers and other stakeholders with different preferences and interests. Another relevant advancement in BDT was made by Tversky and Kahneman (1974) who proposed and verified one hypothesis that threatened normative decisional approaches: decision makers act according to unstable and ambiguous preferences (Slovic et al., 1977). This conceptualization was based on the idea that people have multiple selves with conflicting assumptions, causing them to act inconsistently with regard to their previous choices.

Developments in BDT reinforced the need to enlarge the debate on human rationality to also include unconscious “irrational forces”; in this regard, Simon later added that “to have anything like a complete theory of human rationality, we have to understand what role emotion plays in it” (Simon, 1983; p. 29). Stemming from this last assumption, over the past decades, many scholars (Barsade and Gibson, 2007) have attempted to analyze the impact that both affective and cognitive variables play in the decision-making process. As an example, one recent contribution toward this last direction is the one by Treffers et al. (2020), who demonstrated that managers in a positive affective state and under high time constraints elaborate fewer original and fewer feasible strategic ideas and make their original strategic choices worse when compared with managers in a negative affective state and under high time constraints, who generated better original strategic choices.

However, despite the growth of research on the affect-cognition debate in managerial decision making, there has been a profound division among scholars concerning whether affective states influence cognition or *vice versa*. This can be seen in the debate on the roles of System 1 and System 2 in our mind. Indeed, within dual mind processing theories (Kahneman, 2003), there are two main schools of thought that flourished over time (see Evans, 2021 for a discussion): *(i) default-interventionist*, whereby, as a default setting, individuals make decisions recurring to intuition/emotions and reflective thinking may intervene dependent on the task at hand (e.g., Stanovich and West, 2000); and *(ii) parallel-competitive*, whereby, intuitive/emotional and reflective processes operate in parallel, such that “in the event of conflicts between them, they literally compete for the control of thinking and behavior” (Hodgkinson and Sadler-Smith, 2018; p. 483).

Stimulated by these different visions within dual mind processing theories and strongly anchored in BDT, many contributions have tried to shed light on the relationship between affect and cognition in decision making. For example, Blanchette and Richards (2010) reviewed a series of articles to identify if and how affective states have repercussions on cognitive mechanisms. In particular, these scholars concluded that cognitive biases are mainly linked with anxiety and that (high/low) risk perception is also influenced by (negative/positive) affective states. However, according to them, sometimes affective states hinder normatively correct thinking, while in other cases, they promote it. In the same vein, Lochner and Eid (2016) proved that both negative and positive affective states drastically impact individuals’ reasoning performances.

On the other hand, another group of scholars claimed the supremacy of cognition over affective states. In this regard, Grecucci et al. (2020) recently hypothesized, tested, and verified that cognitive strategies are powerful enough to alter emotional states. Nevertheless, between these two opposite points of view, there is a third group of scholars who assumed that emotions and cognition could not be analyzed separately; indeed, they claimed that the affect-cognition debate should be studied by adopting lenses of mutual interplay since cognitive and affective domains should be perceived as two faces of the same coin (Gosling et al., 2020).

In this last vein, Cristofaro’s (2019; 2020a,b; 2021a,b; Cristofaro and Giannetti, 2021) recent and in-depth contributions rooted in BDT enriched the debate by discussing the role of affect in management decisions, also proposing an *Affect Cognitive Theory* to explain how decision-making processes occur by considering the interplay between affective states and cognition. Hence, this new theory proposes that the crucial circumstances in which emotional states influence/are influenced by cognition and its biases identify that decision makers are affected by multi-level variation of both physical and social scenarios. Under these circumstances, decision makers are perceived as “emotional cognizers”, overwhelming the thinking-feeling dichotomy often promoted in the precedent studies of management decisions.

### Neuroscience in Management and Organization Studies

The first seminal contribution that tried implementing neuroscience techniques/points of view in management studies was by Taggart et al. (1985). In particular, by analyzing the link between decision style and cerebral dominance in 71 subjects by the use of an Electroencephalogram (EEG), these scholars concluded that psychological measurement captures very little actual cerebral processing. In practice, a boost of neuroscience adoption was encouraged to arrive at more solid managerial implications when concerning decision making.

After that stroke of genius, a period of stagnation followed and contributions aimed at connecting the management and neuroscience disciplines started appearing again – but not in a continuous way – only around the 2010s. This raised interest gave light to the field of ‘‘organizational neuroscience’’, aiming at using neuroscience knowledge and approaches at different levels in organizations, as well as promoting linkages to management practice^1^. However, due to the strong epistemological and ontological differences of these two disciplines, there have been many “positioning” contributions oriented to provide a solid direction for this cross-fertilization.

With regard to the above, the work by Laureiro-Martínez et al. (2015a), which discusses the possible merge of cognitive neuroscience and strategic management starting from the value in their complementarities (see also Ascher et al., 2018), is noteworthy. In particular, they suggested three pillars – task selection, sampling, and ethical issues – for a successful mutual implementation of neurosciences and strategic management and provided a research agenda about the several circumstances of synergy between management and neuroscience researchers. Yet, the authors underline the advantage of neuroscience for management research laying in the possibility to scrupulously analyze the decisions made by managers at the brain level, a locus of psychological formation that cannot be intentionally biased by the participant.

In a similar vein, Murray and Antonakis (2018) and Jack et al. (2019) also provided updated suggestions about how to advance this new area of research. Under a methodological point of view, Jack et al. (2019) have envisioned that neuroimaging procedures, particularly functional Magnetic Resonance Imaging (fMRI) and Electroencephalogram (EEG)^2^, are expected to provide a lot of support to organizational neuroscience over the following decades. However, in line with Powell (2011); Jack et al. (2019) highlighted that reverse inference, i.e., inferring the presence of a specific cognitive process from observed brain activation, is mandatory for neuroscience to inform scholars involved in the organizational field consciously. However, as advanced by Murray and Antonakis (2018), the hype and the unfamiliarity with the methods made scholars cautious about adopting neuroscientific methodologies in social sciences. Notwithstanding, in terms of benefits, Murray and Antonakis (2018) have pointed out that neuroscience data are exempt from the “cheap talk” and social desirability that can bias self-reports and surveys (Podsakoff and Organ, 1986). In fact, data coming from neuroscience have several advantages such as their immediate observability, impartiality, and require relatively low-cost measurement tools.

### Affect and Cognition in Neuroscience

Within the last decades, due to the emergence of powerful new tools for assaying the brain, researchers in cognitive psychology and neuroscience have been able to identify and validate the foundations of the decision model (e.g., Fellows, 2004) while looking at affect and cognitive mechanisms.

In particular, neuroimaging studies have identified two main brain regions involved in the “cognitive” system: the Anterior Cingulate Cortex (ACC) and the dorsolateral Prefrontal Cortex (dlPFC). The ACC’s dorsal part is linked with the Prefrontal Cortex (PFC), the Parietal Cortex (PC), the motor system, and the Orbitofrontal Cortex (OFC). In terms of functions executed, the ACC processes top-down and bottom-up stimuli and assigns specific control to other areas of the brain. Regulation of norm enforcement and self-interest, and adaptive response to a changing condition, are juxtaposed with the emotions in this structure (Knoch et al., 2006). The dlPFC is part of the PFC. Yet, the dlPFC has been associated with functions executed as switching attention, working memory, abstract rules, and inappropriate response inhibition (Fehr and Krajbich, 2014).

Regarding the “affect” system, this has been found to include three independent areas known to serve as broad functions in emotional processing, including mind-body integration of affective information and fundamental for experience and expression of emotions: insula, amygdala, and ventromedial Prefrontal Cortex (vmPFC). The insula is a portion of the cerebral cortex folded deep within the fissure, separating the temporal lobe from the parietal and frontal lobes. The amygdala is an almond-shaped set of neurons located deep in the brain’s medial temporal lobe, which has been shown to play a critical role in processing emotions, necessary for triggering aversive emotional states from primary inducers (Haruno and Frith, 2010). The vmPFC is situated in the medial portion of the PFC and has been implicated in various social, cognitive, and affective functions; for example, it is critical for generating and regulating negative emotions and the representation of reward and value-based decision making. This is why a vmPFC study is essential in encoding subjective values of perceived offers and emotion regulation (Gilam et al., 2015).

The described brain areas (see Appendix 1 for a graphical illustration) can also be re-interpreted according to the largely adopted brain categorization of the X- and C-systems (Lieberman et al., 2002). This view includes, in contrast to the dual-process theories, the social cognitive neuroscience perspective, which implies a social interaction to drive behavior. Indeed, the dual system, represented for example by the Kahneman systems 1 and 2, is a more individualistic view, which highlights only the personal perception of the context. Although, the managerial decision making includes the individual along with external data processing from the social context, which reinforces our statement that Lieberman’s framework is adapting to provide a more complete and complex view in which managers need to operate. Environmental, social, and cultural conditions that should not be included in the decision-making process are, instead, part of it due to the inner characteristics of individuals finding they have to choose in the event of uncertainty.

In particular, the X-system is associated with non-conscious environmental analysis, which some scholars have described as automatic processing, implicit learning, and even intuition. In practice, the X-system conducts perhaps a vast majority of everyday processing (Reynolds, 2006). The X-system has many components: the ventromedial PFC (vmPFC), basal ganglia (BG), amygdala (A), lateral temporal cortex (LTC), posterior superior temporal sulcus (pSTS), temporal pole (TP), and dorsal anterior cingulate (dACC) are the most relevant to automatic cognition. On the contrary, the C-system is the mechanism by which complicated reasoning is accomplished (see Lieberman et al., 2002). Specifically, the C-system is capable of rule-based analysis and can be interpreted as a complex analytical tool able to take the facts of a situation and apply an abstract decision rule to determine an outcome (Reynolds, 2006). Yet, when activated, the C-system performs a regulatory role over the X-system. In terms of composition, the C-system is formed by lateral PFC (LPFC), ventrolateral PFC (VLPFC), medial temporal lobe (MTL), medial parietal cortex (MPAC), lateral parietal cortex (LPAC), rostral ACC (rACC), medial PFC (MPFC), and dorsomedial PFC (DMPFC).

However, for the sake of clarity, it is noteworthy to say that despite the apparent clarity and distinctions of brain areas, neuroscience studies also advanced some different positions about the functioning of affective and cognitive mechanisms. For example, Adolphs and Damasio (2001), by reconsidering previous laboratory findings of cognitive neuroscience, highlighted that affect and cognition are inseparable and that the former drives the latter. Specifically, they reaffirmed that the arousal of affective states is the first reaction external stimuli, and that in this process it is the amygdala that rapidly triggers physiological changes in response to emotionally salient stimuli. Thus, through the vmPFC, the influence of affective states on cognition happens through changes in the visceral state (e.g., heart rate, blood pressure, gut motility – somatic markers in general) that then affect cognitive processes (e.g., learning through failures and being aware of the future consequences of decisions). This conceptualization is at the basis of the *somatic marker hypothesis* (Bechara, 2011).

At the center of the somatic marker theory, there is the assumption that decision makers encode the consequences of choices effectively (e.g., Pessoa, 2008). In particular, according to Reimann and Bechara (2010), when making a decision, “the immediate prospects of an option may be driven by more subcortical mechanisms (e.g., via the amygdala) that do not require a PFC. However, weighing the future consequences requires the PFC for triggering somatic responses about possible future consequences. Specifically, when pondering the decision, the immediacy and prospects of an option may trigger numerous somatic responses that conflict with each other (that is, positive and negative somatic responses). The end result, though, is that an overall positive or negative signal emerges (a “go” or “stop” signal)” (p. 770). Therefore, from that theory – that is not an excerpt of criticism (see Dunn et al., 2006) – it emerges that complex cognitive-emotional behaviors are grounded in dynamic coalitions of brain areas’ networks.

In this vein, Pessoa (2008) has deeply highlighted that behavior should be perceived as the result of the mutual interaction of different brain areas, proposing, at the same time, the idea that emotion and cognition not only strongly interact in the brain but that they also jointly contribute to shaping human actions. In particular, Pessoa (2008) has remarked that the amygdala plays an essential role in forming individuals’ emotional aspects, while the PFC is responsible for the cognitive one. However, as recalled by this scholar, several brain regions are loci where both the affect and cognitive mechanisms interact vigorously, such as in the lPFC and the dlPFC. This hypothesis is also supported by evidence on brain structure in highly clustered synapses. Hence, brain areas cannot be considered as watertight compartments. From that, it can be said that Pessoa (2008) was one of the first who advanced that cognitive and affective mechanisms are mutually influenced.

## Methodology

**FIGURE 1:**
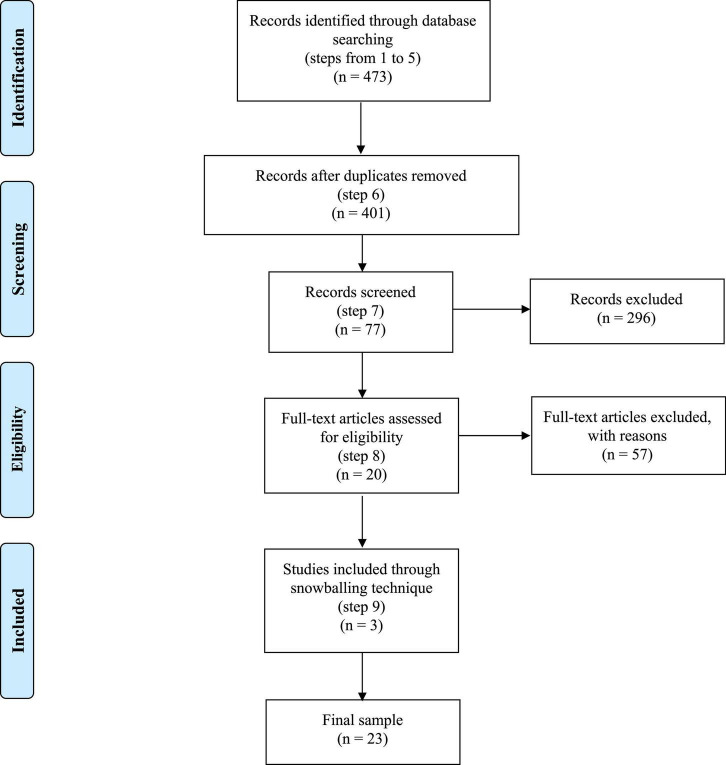
PRISMA representation of papers’ collection strategy.

In order to answer the research question: “*How do affect and cognition interact in managerial decision making?*”, we implemented a Systematic Literature Review (SLR) of contributions dealing with the role of affect and cognition in managerial decisions that adopted neuroscience techniques/points of view. In this regard, we identified the SLR methodology as the suitable research design to consolidate and synthesize academic research. In particular, this method differs from the traditional narrative reviews in: *(a)* assisting in linking future research to the questions and concerns that have been posed by past research, and *(b)* being more explicit in the selection process by employing rigorous and reproducible evaluation methods. In this work, the established SLR procedure by Tranfield et al. (2003) has been followed; see also Figure 1.

(1) The databases for the identification of the studies were: *(a)* Business Source Premier (EBSCO); *(b)* ProQuest’s ABI/Inform; *(c)* ISI Web of Science; *(d)* Scopus; *(e)* PsycINFO; and *(f)* PubMed (including MedLine);

(2) Only peer-reviewed journal articles published in English have been included to enhance quality control. Furthermore, the research was not restricted to a given starting period (end period December, 31th 2021) and type of paper (qualitative or quantitative). These two criteria enhanced the value-added of this work compared to the one by Butler et al. (2016), who just considered empirical papers published between 2007 and 2014.

(3) Only articles adopting a neuroscientific methodology/point of view have been considered by using the keywords: “neuro*” or “brain*” or “Functional Magnetic Resonance Imag*” or “FMRI” or “Electroencephalograph*” or “EEG” or “Magneto Encephalograph*” or “MEG” or “Transcranial Magnetic Stimulation*” or “TMS” or “Transcranial Electric Stimulation” or “tES” or “Positron Emission Tomograph*” or “Functional Near Infrared Spectroscop*” or “fNIRS” or “skin conductance” or “Galvanic Skin Response” or “GSR” or “GRV” or “skin conduct*” or “electrodermal activ*” or “eye track*” or “gaze track*” or “pupillometr*” or “pupil diamet*” or “heart rate*” or “HRV” or “facial expression recognition” or “emotion recognition” or “non-invasive brain stimulation” or “NIBS” or “cortisol” or “testosterone.” These keywords have been derived – and enlarged – following Butler et al. (2016) and Ascher et al. (2018) and aim to identify works related to cognitive neuroscience. 28,705 results were produced.

(4) The substantive relevance of contributions to the managerial decision-making theme has been ensured by requiring that the selected abstracts contained at least one of the following words: “decision*” or “choic*” or “preference*” or “judg*.” These keywords have been derived from the SLR by Cristofaro (2019) on affecting management decisions. 3,070 results were produced.

(5) Only articles regarding business issues have been considered by using the keyword “organization*” and its synonyms: “compan*” or “manag*” or “corporat*,” or “firm*” or “business*” or “enterprise*” or “venture*” or “start-up*” (these keywords have been derived following De Vita et al., 2013). 633 results were produced.

(6) Duplicates from databases were eliminated at this stage thanks to the integration operated through reference-manager software. 401 hits were produced;

(7) The resulting articles were scanned by reading all the abstracts to ensure their substantive context, mainly according to their coherence with the review’s aim. When there was doubt about the content regarding the inclusion/exclusion of an article, the full text was examined. 77 results were produced;

(8) The remaining papers were fully read to ensure their alignment with the research objective. Thus, we included in the sample *only* scientific contributions dealing with managerial decision-making processes that implement neuroscience techniques/points of view; 20 results were produced. In this regard, we implemented the same exclusion criteria as Butler et al. (2016) – see their online S-2 Appendix. Initially, the authors individually read the articles and then compared their evaluations; when disagreeing, the authors assessed the papers together and decided whether or not to include those papers within the sample. Cronbach’s alpha for inter-rater reliability was 0.95.

(9) Snowballing techniques have been applied to the reference lists of the resultant 20 articles. This ensured that important works in the field were included that might have been missed. Three were added arriving a final sample of 23 articles (almost double that of Butler et al., 2016; *N* = 12; yet, these two samples are very different too in terms of selected papers; see Table 1).

**TABLE 1:** The study sample’s papers.

	Author(s)	Year	Type of article	Setting	Methods	Dependent Variable(s)	Independent Variable(s)	Analysis methods	Main findings
1	Srinivasan and Balasubramanian, 2003	2003	Conceptual paper	Neuronal architectural framework	−	−	−	−	As leadership in the emerging millenium achieves new dimensions, sustaining precognition would be most critical. This circumstance would not be fulfilled unless: (1) managers stay anchored to a deeper region of consciousness and make sure all decisions or cognitions emanate from there; (2) there is an effort to acquire complex and new inputs or experiences constantly, so that the brain is primed for incessant change as it ensues. Both these conditions would guarantee that the world economic order in the next millenium stays both robust and customer centric.
2	Wenstøp, 2005	2005	Conceptual paper	Emotion in Multi-criteria Decision Analysis	−	−	−	−	Rationality requires that both beliefs and values be well founded, and values cannot be well founded without emotion. Thus, rational decision making (or emotional rationality) requires elicitation of emotions. However, Multi Criteria Decision Analysis cannot handle virtues well, although questions involving virtues are usually very emotional. Therefore, proper MCDA requires a careful separation of virtues and ends, and then focus on the ends in the subsequent analysis.
3	Reynolds, 2006	2006	Conceptual paper	Neurocognitive model of ethical decision making	−	−	−	−	Explaining, predicting, and motivating ethical behavior are goals worth pursuing. Accomplishing these goals, however, requires models that adequately disclose ethical decision making in a way that sparks research and fosters application. This neurocognitive model is such a model, and the extent to which the authors can expand and apply such a perspective to these uncommonly complex issues gives the authors a greater chance of achieving those goals.
4	White et al., 2007	2007	Empirical paper	166 MBA students	Testosterone measurement	Entrepreneurial experience; family business background	Testosterone level	Logistic regression	This study presents theory and evidence linking the combination of both sociological and biological factors with new venture creation: a biosocial model of entrepreneurship. Empirical results indicate new venture creation is more likely among those individuals having a higher testosterone level in combination with a family business background.
5	Hodgkinson et al., 2009	2009	Conceptual paper	Intuitive and analytical approaches to decision making	−	−	−	−	The rapidly expanding developments in social cognitive neuroscience investigated in this article look set to further corroborate and enhance current understanding of intuition, bringing vital scientific foundations for its increasing role in organizational life as well as a framework of lessons for managers.
*6	*Krueger et al., 2009	2009	Empirical paper	67 United States combat veterans	Computed tomography (CT) scans	Emotional Intelligence	Perception and integration of emotional information	Mayer-Salovey-Caruso Emotional Intelligence Test (MSCEIT); ANOVA; Wechsler Adult Intelligence Scale (WAIS) III; Gaussian distribution (Kolmogorov–Smirnov test); variance homogeneity (Bartlett’s test); non-parametric tests (Kruskal-Wallis test)	This study shows that competencies underlying emotional intelligence (EI) have clear neural foundations and can be impaired despite otherwise normal basic intellectual functioning. Prior findings have indicated that the behavioral and emotional dysfunction associated with vmPFC damage cannot be explained by impaired cognitive intelligence measured by standard intelligence tests. Moreover, although the dlPFC has been correlated with cognitive intelligence, recent lesion evidence failed to endorse the hypothesis that dlPFC damage would disproportionately impair general measures of cognitive intelligence. On the other hand, EI complements cognitive intelligence and permits the assessment of individual discrepancies in emotional and social processes – such processes are key factors in making the right vs. wrong decisions in one’s personal life and in influencing people’s choice about optimal situation-specific social and economic exchange strategies.
*7	*Boyatzis et al., 2012	2012	Empirical paper	7 people, enrolled as senior-level managers, business owners, or second career faculty members	fMRI	Activation of neural areas	Recalling experiences	Least-squares regression	The results showed compelling activation or negative activation of 31 different brain regions for all subjects with 23 of these remaining significant with the exclusion of the single female subject. The findings seemed to cluster in a manner that was puzzling. Because this was an exploratory study, scholars could only define the possible connotations of these findings in light of past research; future studies will be needed to test these interpretations and determine which regions are critical to effective leadership and the role of gender.
8	Linkov et al., 2012	2012	Conceptual paper	Neuroscience and decision making	−	−	−	−	How the brain makes decisions adopting imperfect information is a pivotal question of modern cognitive neuroscience. First, despite its irrationalities and inefficiencies, the brain remains by far the most flexible and complex decision-making tool available and, therefore, may be an appropriate model for structuring decision-making mechanisms, similar to other biologically inspired solutions to real-world problems in computation, optics, immunology, and other fields. Second, policy decisions must basically depend on human judgment and, thus, will be best served by methods and tools that complement human abilities.
*9	*Hannah et al., 2013	2013	Empirical paper	103 military executives	EEG (qEEG)	Psychometric- and Neurologically based measures	Adaptive decision making	Standardized self-complexity measure	The authors have derived psychometric- and neurologically based measures demonstrating that both of them are calibrated for unique variance in external ratings of adaptive decision making. Furthermore, the authors have argued about how these findings can provide a deeper understanding of the latent and dynamic mechanisms that underpin leaders’ self-complexity and their adaptability.
10	Hodgkinson and Healey, 2014	2014	Conceptual paper	Discuss the conditions for a framework that enables firms to harness the cognitive and emotional capacities of individuals and groups	−	−	−	−	As scholars have determined, emotion is pivotal to enabling radical innovation. However, ongoing organizational practices are predicated on a (bounded) rationality façade, rooted in the cold cognition era. This has unintended consequences for organizations, both in respect of formulation and implementation attempts to foster radical innovation.
11	Leger et al., 2014	2014	Empirical paper	21 MBA students	Electrodermal activity (EDA)	IS_ERP; IS_HUM	Non-specific amplitude of electrodermal response (AMP.NS.EDR); Non-specific electrodermal response (SD.NS.EDR)	Descriptive statistics and correlations of the variables	Results show that both expert and beginner users exhibit considerable EDA activity during their interaction with the ERP system, indicating that ERP use is an emotional mechanism for both groups. However, the findings also indicate that experts’ emotional responses led to their sourcing information from the ERP, while novices’ emotional responses led to their sourcing information from other people.
12	Dedeke, 2015	2015	Conceptual paper	A cognitive–intuitionist model of moral judgment	−	−	−	−	Emotions always guided a worker’s cognitive moral decisions. These emotions could make it more or less likely for the employee to comply with the moral rules. Hence, it is in the interest of the organizations to know the emotions that their employees have when they comply or ignore the company’s moral codes.
13	Laureiro-Martínez et al., 2015b	2015	Empirical paper	63 participants with at least 4 years’ experience of making managerial decisions	fMRI	Activation of the brain circuits related to attentional control	Decision-making performance	ROI analysis	This article could contribute to theories at the intersection of control and attention through a focus on attentional control, as the cognitive systems that experienced decision makers use to shift to alternative options. Attention control guides cognition, particularly when there is no predetermined means to achieve goals. Authors have found a positive correlation between the strength of attentional control and decision-making performance.
14	Butler et al., 2016	2016	Review	14 empirical papers on neuroscience and managerial decision making	Systematic Literature Review	−	−	−	The authors have classified three organizational neuroscience clusters that have already made substantial theoretical improvements to management and organizations. Neuroimaging has the capacity to co-locate the cortical substrates that mediate decision-making processes within the brain, and to relate the processes to time. All three clusters are already providing insights into the specific boundaries surrounding the human freedom to act. Clarifying the more precise function of emotions and their regulation in forming a judgment in managerial decision making in different contexts has been a recurring theme. The organizational behavior batch, probably because of the multiple methods that have been adopted, has also been able to analyze how team members function synchronously, and the links between physical traits and leadership.
15	Chen et al., 2016	2016	Empirical paper	60 accountants with at least 5 years of working experience	Eye-tracking	Time spent focused on the financial and non-financial indicators	Strategic Business Unit information; linked or non-linked performance indicators	ANOVA	Authors have found that respondents who look more at strategically linked performance measures are more likely to make decisions consistent with the achievement of their subordinates’ strategic objectives; and, especially, when respondents were aware of the corporate strategy, they have focused more on strategically linked performance measures than on non-linked measures.
16	Cropanzano et al., 2017	2017	Conceptual	A theoretical model combines the use of justice rules to assess events, cognitive empathy, and affective empathy	−	−	−	−	Authors have claimed that deontic justice is an important moral factor for individuals, even when it does not directly serve their self-interest. In this vein, the authors have hypothesized that deontic justice is the result of the intertwined interaction between the neural systems associated with cognitive empathy, affective empathy, and individuals’ ability to evaluate and apply and apply moral rules. This suggests also that organizations should promote the presence of deontic justice as a part of their overall culture, since it enables the generation of ethical behaviors and, thus, pleasant working environments.
17	Ascher et al., 2018	2018	Review	50 scientific studies on neurostrategy	Systematic Literature Review	−	−	−	Authors have pointed out that tools of neuroscience are promising in strategic management, but there is still much misinterpretation about what would be neuroscientific research and behavioral research, and the contribution to these new fields of studies on strategic management lies on a proposition for a better classification of them.
18	McDonald, 2018	2018	Conceptual paper	Intertwined insights from social cognitive neuroscience sustainability management	−	−	−	−	The central thesis of the paper is about the insights from the arising field of social cognitive neuroscience that have academic and practical consequences for challenges facing sustainability management.
19	Lee and Yun, 2019	2019	Empirical paper	178 business students; 43 business managers	fNIRS	Oxyhemoglobin values on DLPFC	Time constraint	Custom-written MATLAB codes; ANOVA	The authors have found that under high time constraints, individuals can have heightened oxygenation and gamma-range EEG activities. The emotional stress that an agent can experience when he or she chooses a moral option is significant and, thus, there is a need for more future research into the emotional well-being of business agents who have to make hard choices.
20	Rybnicek et al., 2019	2019	Empirical paper	44 MBA students	fMRI	BOLD signal	High income vs. low income	ANOVA; ROI analysis	The findings of this study help to validate *need theory* on a neuroscientific level. In fact, results confirm theoretical assumptions upon which that theory is constructed. First, it is shown if and how far different management rewards are perceived as rewarding and may contribute to work motivation. Second, based on these results, the authors have shown that rewards that closely match a person’s needs are seen as more rewarding than rewards that match those needs to a lesser extent. Moreover, the results extend neuroscientific literature by studying management-relevant rewards that have not been studied before.
21	Boone et al., 2020	2020	Conceptual paper	Neuroscience and CEO social values in investments for Corporate Social Responsibility	−	−	−	−	Authors have brought a corollary illustration based on the results of neuroeconomic experiments to suggest that CEOs’ social values, through association with different sequences of neural processing, affect how responsive they are to compensation arrangements and institutional pressures.
22	Massaro et al., 2020	2020	Conceptual paper	Functional neuroimaging as a tool to advance entrepreneurial cognition	−	−	−	−	Scholars present a cross-disciplinary effort to take a step toward bridging entrepreneurship research and functional neuroimaging, arguing that the time is ripe for the progression of a neuroscience-based standard for studying entrepreneurial cognitive processes and linkages to action. The opportunity to objectively assess mental processes unfolding in the brain, associate such processes with behavior, and ultimately generate physiologically informed theories of entrepreneurial cognition are the pillars supporting why and how neuroimaging can complement, challenge, and ultimately, extend current knowledge in entrepreneurship.
23	Fennimore and McCue, 2021	2021	Conceptual paper	A risk-taking model based on the neurobiology of four motivational states (hope, fear, frustration, and relief)	−	−	−	−	Authors claim that financial managers should be able to manage both their reflexive valuations (i.e., Pavlovian learning) and risk preferences (i.e., instrumental learning) in order to learn the new organizational culture and set of risk preferences. Additionally, the authors suggest that it is viable to follow neurobiological patterns of behavior for those who habitually express risk-aversion, punishment sensitivity, and stronger loss valuations for outcomes, since these motivational states may affect how and why decisions are made and, therefore, help to have a greater understanding of the mechanisms behind such short-, medium- and long-term choices.

The asterisk (*) identifies papers that are also present in the sample by Butler et al. (2016).

The selection at points 7 and 8 followed criteria used by Sandberg and Tsoukas (2015); in particular, studies have been included that explicitly: *(i)* aim to contribute to the development of managerial decision making, and *(ii)* apply neuroscience in their research. So, similarly to Butler et al. (2016), we excluded articles “that only briefly highlighted cognitive neuroscience in a cursory way, for example, in a one-line reference to the topic” (p. 546) and that did not use the terms at point (5) in relation to organizational contexts (e.g., waste-water management).

Following Butler et al. (2016), the 23 contributions related to the role of affect and cognition in managerial decisions that implemented neuroscience techniques/points of view have been structured into three clusters. The literature itself (Braun and Clarke, 2006) defined these inductive emergent clusters and reflected the type of decision made by managers. Sample articles categorized into these three clusters have been read by looking at the assumed relationship of decisions with the reflexive (X-) and reflective (C-) systems (Lieberman et al., 2002;

Lieberman-Aiden et al., 2009). This course of action helps build a solid neuroscientific basis for the assumed connections between neuroscience and the affect and cognition of managers proposed in the discussion section below.

## Results

Among the 23 papers in the sample, only three contributions (i.e., Krueger et al., 2009; Boyatzis et al., 2012; Hannah et al., 2013) are present also in the “organizational behavior cluster” of Butler et al. (2016), substantiating originality and novelty in the systematization we propose. The majority of the 23 papers in our sample are conceptual (12; 52%), then 9 empirical articles (39%) and 2 review works (9%) complete the sample. Among these, authors who have published empirical contributions have used many techniques to test their assumptions; the main ones used have been fMRI (40%), EEG or qEEG (20%), and electrodermal activity (13%). Other techniques used for the data analysis have been: Computed Tomography scans (5%), Facial Expression Recognition (5%), Eye-tracking (5%), fNIRS (5%), and Testosterone (5%). With regard to authors, Gerard P. Hodgkinson is the only one present with two contributions (both conceptual).

**FIGURE 2:**
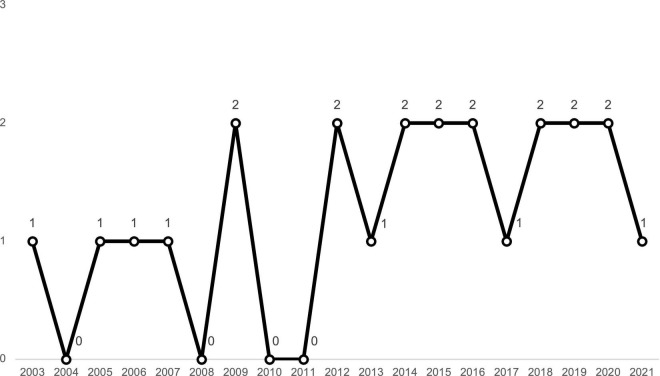
Number of sample papers published by year.

The 23 selected papers cover a period of 18 years, with the distribution shown in Figure 2. There is an average of two publications per year. Furthermore, the selected contributions were published in many different journals; among them, 4 (15%) appeared in *The Leadership Quarterly*.

An inductive thematic analysis of the 23 selected papers’ manuscripts has been implemented, aimed at identifying the type of decisions treated. In particular, thematic analysis is principally employed to acquire a nuanced comprehension of spontaneous and sophisticated processes (Mills et al., 2009), such as decision making. Within inductive thematic analysis, there is no presence of an initial codebook and themes are free to emerge. In line with Strauss and Corbin (1998), the coding has relied on the research question to determine themes associated with the main aspects of the analysis based on the theoretical background. The resulting themes/decision types emerging from the manuscripts dealing with managerial decision making are: *(a)* ethical decisions, *(b)* innovation decisions, and *(c)* data-enabled decisions.

### Ethical Decisions

Our systematic review includes many publications on ethical decision-making processes (i.e., consistently evaluating and choosing ethical principles), emphasizing moral aspects. In this vein, Reynolds (2006) was the first to report the interaction of the C- and X-systems in the ethical decision-making process. In particular, according to Reynolds (2006) model, ethical decisions come from the accuracy of the neural pattern of the stimulus, i.e., prototype, which are activated in the brain of the decision maker, such that: “an effective and thorough search can facilitate ethical behavior by gathering enough accurate information either to match a prototype correctly or to apply the moral rules available effectively” (Reynolds, 2006; p. 743). Prototypes are evoked according to the information *reflexively* collected and categorized into ethical patterns by the decision maker. The ability to structure information to match multiple prototypes will be positively associated with ethical behavior. Then, the ethical judgment is made, and it would be highly positively correlated to ethical behavior if performed by the C-system rather than by the X-system. This last avenue allows restructuring prototypes in the light of a rationalization process of moral rules and, in sum, substantiates that the C-system would exert control over the X-system to ethically manage organizations.

However, research has reported that not only the pre-frontal cortex, but also the amygdala (involved in memorizing emotional reactions) are important for a person’s moral development (Greene, 2015), suggesting that “the discussion of ethics should not rest solely on a rational decision-making model” (Robertson et al., 2017; p. 691). This adheres to the conceptualization of moral development by Kohlberg and Hersh (1977) that does not consider moral judgment as a necessary condition for moral action, because of the fact that also emotions, and a general of sense of will, come into play. Therefore, moral reasoning does not always lead to moral behavior. In this vein, Dedeke (2015), in his theoretical model, suggests that ethical decision making includes five interdependent yet functionally distinct steps and proposes an intuitive view of ethical judgment, as it describes how emotion regulation, perceived moral intensity, and perceived ethical climate constructs impact the formation of moral intent (Dedeke, 2015). In particular, Dedeke (2015) proposes that emotions always influence employes’ cognitive moral decisions. He assumes that automatic cognition (i.e., intuition) and automatic emotions interact within the pre-processing stage of an ethical decision. This would happen as follows: *(i)* the situation faced by the decision maker elicits some memories (as knowledge structures, schematic mental structures) that recall actions that have been implemented in the past and that can also have a role in ethical decisions; and *(ii)* concurrently, emotions provide a frame of reference for cognition; emphasizing some elements of the context over others. Moreover, the emotional reaction is used as an information point in decision making and works as a driver for cognition (i.e., somehow, the X-system drives the C-system).

In the same vein, McDonald (2018) underpins the importance of social cognitive neuroscience in sustainable management and related ethical decisions. The author reports several research insights from the literature, underscoring the essential role of social cognitive neuroscience in corporate sustainability management research. In particular, McDonald (2018) focused his attention on managers and their responsibilities on creating value in an integrated manner across ecological, economic, and social spheres. In doing that, he highlighted the importance of the amygdala, which was found to be assisting emotional learning in ethical decision-making processes. According to this author, the amygdala allows bypassing the cortex providing an automatic and unconscious reaction (X-system over C-system) in unforeseen situations and humans respond immediately to an input, such as fear. To improve ethical decisions in sustainability management, this latter needs to be communicated so that emotional tags are created within the memory to evoke a future state that will facilitate creative solutions. In this regard, the X-system of decision makers, in which the amygdala operates, should prepare the field for an oriented cognition operated by the C-system. This is aligned with the results by Lee and Yun (2019) who identified, using fNIRS, an increase in the hemodynamic responses in the dlPFC that can be linked to moral stress, caused by time constraint and that causes shifting to a proself-condition (i.e., adopting a selfish behavior). Accordingly, it could be hypothesized that activated dlPFC correlates with the capacity to handle moral stress, and can easily affect the C-system favoring the X-system in ethical decision-making processes.

Fennimore and McCue (2021) also deepen the role of stress and other motivational drivers in ethical decision making. From their model, decision makers are neurobiologically inclined to be engaged in risk-averse behaviors once they are persuaded by the fear of disrupting the *status quo* and seek relief by preventing punishment. Yet, other decision makers have neurobiological preferences toward risk-seeking behaviors, galvanized by the hope of reward prospects. In brief, from this study, the X-system seems to orient the risk orientation behavior (similarly to Cropanzano et al., 2017), who studied the neurobiological origins of deontic justice; the moral obligation to uphold norms of justice. In particular, Cropanzano et al. (2017) pointed out that the presence, or absence, of business ethics within the organizational environment are likely to affect positively, or negatively, the behaviors of those involved, since “the pernicious effects of injustice are likely to be spread rapidly through an organization, as some employees become displeased with the treatment and experiences of their coworkers” (Cropanzano et al., 2017; p. 746). Consequently, in deontic justice contexts, the emotional state (X-system) seems to prevail over the rational one (C-system) for the formation of ethical choices.

### Innovation Decisions

With regard of innovation decisions, i.e., the choice to adopt or not adopt an innovation, Hodgkinson et al. (2009) identified intuition as crucial for those organizations seeking innovation, such as new business opportunities (i.e., exploration). Hodgkinson and Healey (2014), demonstrated that reflexive processes (associated with the X-system) are not relegated to a mere source of error or bias to be overcome with effort; rather, they are integral to reflective (C-system) processes of human cognition and critical for skilled processes such as intuition. In particular, they added that, to achieve a successful level of innovation and shifting strategic choices, managers must be offered the opportunity to regulate their feelings (the so-called emotional reframing); adaptive regulation stimulates the PFC.

Such adaptability may be contingent upon managers having the requisite complexity to facilitate effectiveness across various roles with different grades of intuition and expertise. Hannah et al. (2013) specifically examine managers’ self-complexity, which is based on the self’s central role in managing the interface between a manager’s internal processes (closer to the C-system) and his or her interactions with the social environment (closer to the X-system). More effective managers possess a requisite level of complexity that allows them to perceive and assess complex and changing dynamics accurately and, in turn, adapt their decision making and behaviors to enact effective responses. This was confirmed by these scholars through the measurement of the executive and cognitive-associated frontal lobe by quantitative EEG. In addition, Hannah et al. (2013) have recommended that practitioners assess managers’ self-complexity (LSC) to measure their ability to handle internal processes – i.e., goal system, self-regulation, and identity – and their synergies with the external environment. Specifically, Hannah et al. (2013) have highlighted that LSC is a reliable precursor of managers’ adaptive behavior; thus, according to these scholars, LSC can be spotted in managers that can lead them to achieve goals characterized by a higher degree of complexity.

To confirm what was proposed by Hannah et al. (2013); Laureiro-Martínez et al. (2015b) have used the fMRI technique in a sample of expert decision makers that exploitation – deepening the existing business – relies on brain regions (i.e., the mPFC and the hippocampus bilaterally) mainly associated with anticipation of rewards, while exploration – looking for new business opportunities – depends on regions primarily associated with attentional control (bilateral parietal and frontal regions, e.g., dACC). From that, exploitation and exploration are separate behaviors involving different mind processes. Moreover, Laureiro-Martínez et al. (2015b) found that the locus coeruleus-norepinephrine (LC-NE) system and the PFC are activated by the cognitive processes that enable decision makers to switch between exploitation and exploration processes. In particular, these brain regions can be traced back to the C-system and, thus, the LC-NE, which controls the degree of attention, affects this particular system rather than the X-system. In practice, brain circuits related to attentional control allows individuals to achieve better decision-making. Nonetheless, the rational and attentive management of exploration-exploitation processes usually leads, as for March (1991), to selecting more reliable business actions, such as exploitation, rather than those leading to uncertain outcomes – thus, degrading, however, organizational learning in a mutual learning situation and compromising the competitive position in the long term. In brief, even if the more attentive brain processes are put into action, they cannot ensure that the produced equilibrium between exploration and exploitation is the best for successfully adapting to the changing environment.

### Data-Enabled Decisions

Some neuroscience contributions (Damasio, 1994; Bechara and Damasio, 2005) intensely stressed the idea that decision-making processes are not exempted from being affected by emotions and, in recent times, scholars started investigating the emotional responses of decision makers in data-enabled decisions (i.e., decisions facilitated by information technology (IT) systems and related produced data). In this regard, pioneers such as Dimoka et al. (2011) asserted that, in different social contexts in an office environment, exploring the potential of cognitive neuroscience and information systems (neuro-IS) research offers examples of a fertile intersection in which there is a considerable potential to optimize management activities.

Following this line of research, Leger et al. (2014) measured emotional responses, based on electrodermal activity, in two samples of managers with different expertise during the use of an enterprise resource planning system in a decision-making context led to different sourcing information. These scholars found confirmation that the more people become proficient in doing a specific task, the higher the decreasing activity in the prefrontal brain regions. Moreover, as the prefrontal brain regions are correlated with the cognitive side, it can be hypothesized that the more a person becomes confident with a task, the more intuitive his/her behavior will be and the X-system will prevail over the C-system. Hence, remarking on the contribution brought by Krueger et al. (2009) concerning the role covered by emotional intelligence (i.e., the ability of reasoning about emotions, and, in turn, to use emotions to intensify reasoning) in driving individuals’ reasoning and behavioral skills, Leger et al. (2014) shed light on the function played by emotions in IT systems’ frameworks – marking the distinction between proficient and neophyte users.

Similarly to Leger et al. (2014), through the means of the Locarna eye tracker, Chen et al. (2016) investigated how decision quality is affected by the amount of time managers spend looking at the Balanced Scorecard (BSC)’s performance metrics, and whether understanding of a firm’s strategy and how the presentation format affect individuals’ focus. In this context Chen et al. (2016) found that: *(i)* managers who look more at strategic performance measures (e.g., sales margin, brand recognition rating, and employes’ satisfaction) are more likely to make decisions consistent with the achievement of their subordinates’ strategic objectives, and *(ii)* when managers are informed about the strategy put in place by their organizations, they are more concentrated on strategic performance measures than others. Therefore, it could be possible to conjecture that the C-system overrides the X-system in shaping managers’ approach to the results depicted in the BSC.

## Discussion

Generally speaking, the distinction between the X- and C-systems, building upon the lateralization of the brain, although valid, is a coarse classification of many distinct human faculties that pertain to one of two broad domains and their interaction. This general distinction oversimplifies brain lateralization and assumes absolute functional differences in management studies. Our literature review consistently shows that the distinction between reflexive and reflective systems dominates the debate with marginal articulations within each of the systems (Laureiro-Martínez et al., 2015a,b; Boone et al., 2020). In this regard, we recognize from our SLR that three different schools of thought emerged: *(i)* the C-system has a predominant role over the X-system; *(ii)* the X-system has a predominant role over the C-system; *(iii)* the C- and X-systems interact, and neither of the two has a primacy.

For the first cluster, Reynolds (2006) study is among the few important works. Indeed, he was the first to report the interaction of the C- and X-systems in the ethical decision-making process. Ethical judgment would be highly positively correlated to ethical behavior if performed by the C-system rather than by the reflexive one. Hence, according to Reynolds (2006), the C-system exerts control over the X-system for the management of organizations. Recently, Boone et al. (2020) conducted a study to assess the pivotal importance of the controlled C-system over the automatic X-system for pushing Top Management Teams (TMTs) to invest in corporate social responsibility.

With reference to the second cluster, many other scholars, instead, have devoted their efforts to analyzing the influence that the X-system has on the C-system. Among them, it is noteworthy to recall the contribution brought by Leger et al. (2014), who, using electrodermal activity to measure psychophysiological responses elicited by arousal, have confirmed what was previously proposed by Krueger et al. (2009), thus that the more a person becomes confident with a task, the more intuitive his/her behavior will be and, therefore the X-system will prevail over the C-system. This latter idea also has found fertile support from Antonakis and Dietz (2010), who have asserted that the decision makers’ emotional intelligence, combined with other elements such as the working memory and the intelligence quotient, plays a pivotal role in shaping leaders’ answers in critical situations, such as when they face constraints in their own cognitive resources. Particularly, leaders’ ability to handle emotional intelligence allows them to be engaged in higher quality decision-making processes since, due to the aforementioned skill, they are more competent in the creation of proactive environments where a contamination of ideas is widely supported and positively perceived (Antonakis et al., 2009).

A third cluster supports the idea that there is a mutual influence between the C- and X-systems. Among them, we recall the important works by Hodgkinson et al. (2009) and Hodgkinson and Healey (2014), proposing a theoretical framework in which the two systems of human mind have close interaction and mutual influence. According to this third cluster, lateralization of the brain appears to be more complex than how it is popularly investigated (Toga and Thompson, 2003). As suggested by Hodgkinson et al. (2009): “old models based on a simplistic left brain/right brain dichotomy are giving way to more sophisticated conceptions, in which intuitive and analytical approaches to decision making are underpinned by complex neuropsychological systems” (p. 277).

From the above, we should be aware that the traditional distinction between reflexive (X-) and reflective (C-) systems, considered alone, cannot be framed as a complete theoretical framework as the nature of the interplay defines specific paradigms. Indeed, many theories assume dual processing of information, but they radically differ in their articulation. A fundamental distinction, mainly discussed in literature with reference to System 1 and System 2, is between a *parallel-competitive* and a *default-interventionist* approach (Evans, 2021). In this last regard, from the analysis of the sample contributions described above, it can be derived that the managerial decisions result as the product of an emotional-driven dialectic of affect and cognition (e.g., Damasio, 1994; Sadler-Smith, 2016; Abatecola et al., 2018; Cristofaro, 2020a,b, 2021a,b), redirecting the discussion on information processing from dual-mind processing theories (e.g., Stanovich and West, 2000; Hodgkinson and Sadler-Smith, 2018) to a “unified” mind processing theory (Sadler-Smith, 2016) for which the two systems of our mind are not in conflict and for which affective states have an initial (but not exclusive) primary driving role. As a consequence, the recent affect-cognitive interplay emerges, under a neuroscientific point of view, as supported, and may be considered as the fertile ground from which a renewed understanding of managerial decision making can move forward – also because its explanations are intertwined with other relevant streams of research such as the Upper Echelons Theory (Hambrick and Mason, 1984; Abatecola and Cristofaro, 2020) and Behavioral Strategy (Powell et al., 2011; Sibony et al., 2017; Abatecola et al., 2021; Cristofaro and Giannetti, 2021). In particular, it seems to be that the provided understanding supports the recent Affect-Cognitive Theory of management decisions by Cristofaro (2021a); in fact, assumptions of this theory clearly identifies an interplay of affect and cognition, with affective states having an initial (but not exclusive) primary driving role, for the formation of choices supporting the cited “unified” mind processing theory.

## Limitations of the Research Field and Future Research

Neuroscience can help to deconstruct and reformulate from scratch some traditional problems – i.e., the roots of behavioral strategy (Powell et al., 2011) – that connote the agenda of management studies. Notwithstanding such premises, the contributions present in our literature review seem not to follow this trend. Indeed, they are characterized more by a mere integration of neuroscientific methods than a radical reformulation of research questions based on neuroscientific evidence.

The main limits of the papers in our sample are that: *(a)* neuroscientific studies are often conducted on non-representative populations, because studies on practitioners are limited, and nothing can grant that the evidence found on non-representative populations (e.g., students) can be generalized; *(b)* when practitioners are present, samples are often limited or biased, as there is difficulty in balancing the different profiles of participating organizations and teams; and *(c)* studies are often based on laboratory experiments.

The above limitations are commonly connected with the intrinsic difficulty of implementing several neuroscientific tools in ecological conditions (e.g., fMRI outside a medical hospital center) and ethical problems in adopting them. In this regard, as expressed by other scholars (e.g., Tivadar and Murray, 2019; Zwaan et al., 2019), since managerial decision-making processes are filled with strong interplay between affective and cognitive contents, the use of either laboratory experiments or non-representative populations will unquestionably lead to results affected by a lower ecological validity and, in turn, to a lesser practical utility of these results to help scholars in the study and explanation of peculiar situations (e.g., by controlling, in real-time, the neurobehavioral mechanisms affecting executives’ decisions while they have to counteract sudden organizational/financial crises).

Furthermore, the neuroscientific contributions examined in this study often present limitations concerning the statistical analysis techniques. More specifically, the number of significance tests carried out in neuroimaging analyses (e.g., fMRI, EEG, and qEEG) are extremely likely to inflate the risk of Type I error (false positiveness) – in line with Jack et al. (2019). A clear example of limitations regarding the statistical methods has been explicitly declared by Balthazard et al. (2012) who have not correctly applied multiple comparisons in their analyses, because this “would impose an overly conservative and impractical limit for exploratory studies like our own” (p. 255). To overcome this limit, Balthazard et al. (2012) have wisely improved the likelihood of any “spurious results” by the means of a second population to replicate their results. Therefore, despite having cross-validated their findings to overcome statistical limitations, this represents a common and evident barrier that many other contributions have had to face.

### An Agenda for Future Research

#### Articulating the Reflexive System

The general distinction between reflexive/X-system and reflective/C-system dimensions seems to substitute the further distinctions among specific cognitive phenomena within the reflexive realm. Affect, intuition, insight, instinct, are quite different types of cognitive phenomena – pertaining to the reflexive system – each markedly connoted by distinctive features and neurophysiological systems. What seems to emerge from our literature review is that such a fine-grained distinction is never by scholars. With a few exceptions (such as Hodgkinson et al., 2009), the selected contributions rely on a coarse-grain distinction between reflexive and reflective systems, avoiding further articulations within each one of them. This theoretical choice seems to discharge the many advancements made in cognitive neuroscience in the last two decades, which tend to articulate on a neurophysiological level of the specific sub-systems involved.

Generally speaking, this review easily indicates that many reflexive processes do not necessarily present an affective dimension. For instance, numerical cognition involved in the well-known “bat and ball” like problems (e.g., Branas-Garza et al., 2019) relies on automatisms but does not consider any affective dimension. What seems to be relevant here is not that distinctive cognitive phenomena are all ascribed to the reflexive system, but they are quite different types whose specificities are underexplored by management scholars.

Assuming a strict functional specialization and rigid modularity between reflexive and reflective systems could be misleading, as it hides several alternative views of the human brain that have emerged in the last decade. System 1 is quite flexible and content-sensitive, as different specialized brain regions are able to contingently interact to form coalitions of brain areas to perform new tasks, instantiating *neural reuse* (Gallese et al., 2021; Mastrogiorgio et al., 2022). Such considerations about the nature of the X-system are quite absent from the contributions that emerged in our literature review and should be explored further in future studies. In particular, and contrary to the idea that the reflexive system is biased, the automatic response can be a source of satisficing decisions in specific task environments (Gigerenzer, 2007). For instance, the recognition heuristic is based on the idea that if one of two objects is recognized, we can infer that the recognized object has a higher value than the criterion to infer it. Generally speaking, organizations that incorporate the affective dimension in decision making are more successful than those organizations that rely solely on analytic approaches (Hodgkinson and Healey, 2011).

The contribution of our literature review seems to acratically assume (with a few exceptions like Hodgkinson et al., 2009) a Manichean duality to overlook the idea that automatic response can generate rational outcomes, which is another avenue for future research. The reflexive system can be a significant source of correct judgments; Hodgkinson et al. (2009) discuss the distinction between insight and intuition, both characterized by the automatic response, where the anterior superior temporal gyrus region of the right hemisphere is related to insight and the orbitofrontal cortex and the amygdala are activated in intuitive judgments (Volz and von Cramon, 2006). If we admit that the reflexive system is a source of rational judgments, we should be tempted to criticize most of the arguments presented in the contributions that tend to unwittingly adopt a sharp distinction between the sources of rational or irrational judgment. We think that continuing the deconstruction of the distinction between irrational/automatic vs. rational/deliberate is a fundamental domain of future research.

#### Articulating the Ecological Dimension

The traditional perspective of *cognitive biases* (Kahneman et al., 1982; Stanovich and West, 2000) has been, in the last decades, complemented by an alternative program emphasizing that decision makers are able to deal with complex environments through the use of fast and frugal heuristics that are adapted to the structure of the environment (Gigerenzer and Selten, 2002). Kahneman’s (2003) framework has been criticized for a poor specification of the role of the environment when formulating judgment, as Kahnemanian heuristics are assessed using non-ecological benchmarks (such as logic and probability calculus) (e.g., Gigerenzer and Murray, 1987; Gigerenzer, 1991). Within the framework of bounded and ecological rationality, it is impossible to assess human rationality only by looking at the cognitive phenomenon, limited-to-the-brain, as the structure of the environment specifies which cognitive process is successful (Gigerenzer and Selten, 2002). Interestingly, the contributions in the sample, despite they have been selected with specific reference to Simon’s (1947) tradition, neglect the role of ecological dimension and, in particular, the fit between cognitive resources and environmental structure (also known as the scissors’ argument, Newell and Simon, 1972). The so-called naturalistic decision making – which analyzes how experts make decisions in ill-structured, complex environments under conditions of time pressure (Zsambok and Klein, 2014) – introduces an alternative perspective on reflexivity and represents a promising, but somehow underexplored, program of research. Organizational neuroscience represents a “natural” articulation of the ecological dimension, where the workplace represents the “real-world” in which specific neuro-cognitive mechanisms can be studied.

#### Views of the Brain and Affective-Cognitive Interaction

Traditionally, experimental research favors a reactive view of the brain, as brain functions are studied by means task-evoked responses. The experimental perspective, though successful, leaves aside the factual consideration that brain activity is mainly intrinsic and involves functions for interpreting and predicting environmental instances, and not just reacting to them. Generally speaking, what makes the study of the *intrinsic brain activity* relevant is that the brain’s enormous energy consumption is not related to specific tasks, but to its default activity mainly related to the ongoing, perceptual information processing of large amounts of sensory data (Raichle and Snyder, 2007; Raichle, 2010).

Although the idea that the brain is not primarily reactive – a default activity occurs prescinding from the responses of contingent tasks – is not new, the investigation of intrinsic brain activity (i.e., “baseline”) represents a relatively under-investigated domain of neuroscientific research, also considering its related methodological problems. Indeed, while experiments are rigorously designed (stimuli and responses can be measured with great precision), the measurement of the default activity of the brain can be elusive, as there is no specific theoretical focus. We think that the role of the intrinsic brain represents a future domain of investigation for decision-making research, which is able to shed new light on the affective-cognitive interplay. As shown in Table 2, while in the reactive brain perspective, articulating the reflexive system and the ecological dimension (discussed in the previous section) represents the two domains respectively related to the affective and cognitive dimension, in the intrinsic brain, the focus is on the long-term default mode of brain functioning. In particular, with reference to the affective dimension, personality is reflected in the brain’s intrinsic functional architecture, where the resting-state functional connectivity is predicted by specific personality traits (Adelstein et al., 2011). With reference to the cognitive dimension, when humans have ample time at their disposal to make a decision – and this is expected in upper echelons contexts – spontaneous brain activity constrains the selection of solution strategy (Barnes et al., 2014), as intrinsic activity may reflect a memory system represented by an internal statistical structure of the outside world (Sadaghiani and Kleinschmidt, 2013).

**TABLE 2:** A potential typology of future research.

		Type	
		Affective	Cognitive
***View of the brain***	**Reactive brain**	Articulating the reflexive system *Biased or “gut feelings”?*	Articulating the ecological dimension *Which cognitive process for which task-environment?*
	**“Intrinsic” brain**	Understanding the role of persistent traits *How do personality traits enter into the process?*	Understanding the “baseline” of expertise *How does intrinsic activity affect expert decision-making?*

The role of the default mode in the affective-cognitive framework helps us to uncover the long-term, stable, not contingent, boundary conditions of decision making. Emerging domain of investigation could be central in organizational neuroscience which, by definition, focuses on the stable “default-experience” of individuals in structured organizational contexts. Notice, incidentally, that this focus on stable default-mode experience can be also useful to investigate the cultural issue (as we expect that specific organizations’ practices enter into the “baseline” in order to affect the way in which decisions are made).

## Implications and Conclusion

*How do affect and cognition interact in managerial decision making?* This is the research question that we tried to answer through the SLR of contributions produced on managerial decision making, which consider neuroscience techniques/points of view. In terms of originality, this is the first contribution filling this gap, stemming from the fact that the only other SLR produced (Butler et al., 2016) did not deeply focus on managerial decision making.

Results of the sample papers show alternative views about the X- and C-systems that seem differently devoted to non-conscious and complicated reasoning. Selected works are not unanimous, but, from their systematic analysis, it can be advanced that the relationship between affective states and cognition is dialectical, with affective states having a driving role toward cognition: the X-system initially drives the C-system. This is aligned with brain studies that point toward a driving role of affective states, since they come from the biochemical response of individuals to their context. In this regard, seeing the relationship between System 1 and System 2 in managerial decision making as parallel, reorients the discussion on information processing from the tradition of sequential dual-mind processing to a “unified” mind processing theory for which the two Systems are not in contrast and for which affective states have an initial (but not exclusive) primary driving role (e.g., Damasio, 1994; Cristofaro, 2020a,b).

From this work, the relationship between BDT – including its developments – and neuroscience emerges as stronger, because one modifies/reinforces the other in a virtuous scientific debate. However, as advanced by Powell (2011), management scholars must not forget that neuroscience can add significant value to the current state of the art in management research only if the former is “at the service” of the latter. Otherwise, neuroscience results could be not perceived as relevant for practitioners and for management scholars themselves, reducing the communication power of the neuroscience-management duality in decision-making research. To avoid that, traditional managerial problems must be re-articulated through a neuroscience lens. Brain imaging techniques can reveal the specific brain area involved in specific decision-making domains. But, there is more. The investigation of intrinsic brain activity could represent a future domain of organizational neuroscience research in which the default-mode of brain functioning can be considered as a “boundary condition” for decision making. This trend is also favored by the increasing use of dedicated devices (such as a stress bracelets, EEG, etc.) in organizational settings. Measuring the default-mode parameters of brain activity can inform a next-generation of practitioners on how to improve decision making.

Our study also shows that real novelty in hypothesis generation, informed by cognitive neuroscience, is somehow missing. While neuroscience allows deconstructing consolidated categories – to reformulate old management problems in a fresh manner – this is not what our literature review shows; instead of generating radically novel hypotheses, it seems that management scholars are more prone to use novel neuroscientific tools to investigate old management problems. For example, with reference to innovation decisions, the dynamics that lead to innovation are framed within the traditional paradigms such as organizational myopia (Laureiro-Martínez et al., 2015b) or managers’ adaptability (Hannah et al., 2013). Interestingly several neuroscientific accounts of innovation-related problems – such as technical reasoning and technological culture (e.g., Osiurak and Reynaud, 2020) – are not explored. This type of consideration also applies to the data-enabled decision, where neuroscientific tools are used in an ecological setting that involves data manipulation (e.g., Leger et al., 2014 measured electrodermal activity during the use of an enterprise resource planning ERP system). Interestingly, despite their neuroscientific claims, such contributions seem to ignore a relevant tradition of neuroscientific evidence dealing with related problems (such as manipulating numerical magnitudes and formats, e.g., Kadosh and Walsh, 2009). Generally speaking, management scholars are more and more prone to neuroscientific investigations. Still, their hypotheses do not seem to be well-informed by the art of cognitive neuroscience and its related debates.

However, if we think that moving from consolidated neuroscientific evidence and debates can be the solution to a solid generation of hypotheses in management studies, we are wrong. Cognitive neuroscience is a dynamic domain characterized by different theories and views of the brain that are often incommensurable. This is particularly true for affect and cognition that, far from being distinct domains, have been shown entangled as cognition is affectively modulated (Damasio, 1994; Adolphs and Damasio, 2001). What should guide future research is the awareness, informed by neuroscientific evidence, that the interplay between affect and cognition could be radically different from what a folk approach could suggest.

In terms of practical implications, managers should take into consideration that their decisions are the concurring product of affective and cognitive influences, with the former having an initial (but not exclusive) role. In this regard, decision makers’ course of action can be regulated acting on the perceived affective state; e.g., decision makers interested in enhancing accurate analyses for a choice should consider planning them after recognizing the dramatic impact that a wrong decision may have – this can be done by referring to the “pre-mortem” technique of Klein (2007) aimed at discovering why a project may fail – so as to insert a negative mood. From what has been said, it also suggested investigating the emotional side (by using, for example, the Positive and Negative Affect Schedule questionnaire) of potential collaborators – at all levels – for a complete evaluation of their decision-making processes. Yet, as advanced many times in this study, one of the biggest limitations of the empirical studies in this field lays in the scarce possibility to accomplish ecological testing of the neurobehavioral processes that shape managers’ decision-making choices. Consequently, this has prompted scholars to rely mainly on laboratory experiments or non-representative populations reducing the validity – given the absence of important stressors (e.g., the awareness that their choices, being taken in the laboratory, will not affect the safety or stability of their organization) – and, in turn, the practical utility of the resulting insights. Therefore, in addition to having a higher confidence toward neuroscience, as well as an improved availability of the instruments belonging to this scientific field, it is desirable to reach greater synergy between scholars and practitioners in order to produce more complete, trustworthy, and meaningful understandings of the real neurobehavioral processes that affect managerial decision-making outcomes, thus resulting in a mutual win. Doing that ensures following the recommendations by Powell (2011) in using neuroscience as the mean to explore behavioral assumptions of managerial decision making, reinforcing, in turn, behavioral strategy research (Powell et al., 2011).

## Data Availability Statement

The original contributions presented in the study are included in the article/supplementary material, further inquiries can be directed to the corresponding author/s.

## Author Contributions

MC and APM equally contributed to section introduction. APM contributed to the sections: neuroscience in management and organization studies, and ethical decisions. MC contributed to the sections: affect and cognition in decision making, methodology, data-enabled decisions, and discussion. PG contributed to sections: affect and cognition in neuroscience, results, innovation decisions, and limitations of the research field and future research. AM contributed to the section: an agenda for future research. MC and AM equally contributed to the section implications and conclusion. All authors approved the submitted version.

## Conflict of Interest

The authors declare that the research was conducted in the absence of any commercial or financial relationships that could be construed as a potential conflict of interest.

## Publisher’s Note

All claims expressed in this article are solely those of the authors and do not necessarily represent those of their affiliated organizations, or those of the publisher, the editors and the reviewers. Any product that may be evaluated in this article, or claim that may be made by its manufacturer, is not guaranteed or endorsed by the publisher.

## Footnotes

1 This definition is taken from the Organizational Neuroscience interest group born within the Academy of Management (https://neu.aom.org/home).

2 fMRI and EEG are two popular non-invasive techniques used in medical sciences to analyze brain responses to specific stimuli evoked by sensory, motor or cognitive tasks in controlled environments. Specifically, EEG uses electrodes to quantify the dynamics of the global neuronal activation with sub-millisecond resolution, while fMRI, by the means of a magnetic resonance machine, produces images of local changes in cerebral blood oxygenation, which are indirect consequences of neural activity. Therefore, given this complementarity – direct vs. indirect analysis of neuronal activation – fMRI and EEG techniques are often used simultaneously.

## Appendix 1

Adapted from Lieberman (2007). Social cognitive neuroscience: A review of core processes. *Annual Review of Psychology*, *58*, 259–289.
